# p21-activated kinases (PAKs) regulate FGF1/PDE4D antilipolytic pathway and insulin resistance in adipocytes

**DOI:** 10.1016/j.molmet.2025.102210

**Published:** 2025-07-12

**Authors:** Judith Seigner, Johannes Krier, David Spähn, Leontine Sandforth, Judith L. Nono, Robert Lukowski, Andreas L. Birkenfeld, Gencer Sancar

**Affiliations:** 1 Institute for Diabetes Research and Metabolic Diseases of the Helmholtz Center Munich, Tübingen, Germany; 2 Department of Internal Medicine IV, Division of Diabetology, Endocrinology and Nephrology, University Hospital of Tübingen, Tübingen, Germany; 3 German Center for Diabetes Research (DZD), Munich-Neuherberg, Germany; 4 Cardiovascular Medicine and Sciences, King's College London, London, UK; 5 Institute of Pharmacy, Department of Pharmacology Toxicology and Clinical Pharmacy University of Tübingen, Tübingen, Germany

**Keywords:** Lipolysis, Insulin resistance, Type 2 diabetes, FGF1/PDE4D, p21-activated kinases, Adipogenesis

## Abstract

Increasing evidence suggests that adipose tissue plays a key role in the development, progression, and treatment of the globally epidemic disease type 2 diabetes (T2D). For example, adipose tissue dysfunction, lipotoxicity, and insulin resistance (IR) are major contributors and targets for the treatment of T2D. We previously identified the Fibroblast growth factor 1 (FGF1)/Phosphodiesterase 4D (PDE4D) pathway, which lowers plasma glucose concentration by suppressing lipolysis in adipose tissue and ultimately regulating hepatic glucose production in obese insulin-resistant mice. While phosphorylation of PDE4D is critical for its activity, the upstream signaling mechanisms remain unclear. In this study, we identified p21-activated kinases (PAKs) as regulator of PDE4D phosphorylation and suppression of lipolysis by FGF1. Inhibition of PAK-induced cAMP accumulation prevented antilipolytic function of FGF1, and reversed suppression of lipolysis caused by PDE4D overexpression, linking PAKs to the regulation of cAMP by PDE4D in murine adipocytes *in vitro*. Chronic inhibition of PAKs decreased lipid accumulation in both mouse and human adipocyte cultures, lowered expression of adipogenic markers, and induced IR, suggesting a previously unidentified role of PAKs in adipocyte function and differentiation. We conclude that PAKs play a crucial role in regulating the FGF1/PDE4D antilipolytic pathway, adipogenesis and IR, thereby highlighting their potential as therapeutic targets for T2D.

## Highlights

• p21-activated Kinases (PAKs) regulate Fibroblast growth factor 1/Phosphodiesterase-4D dependent suppression of lipolysis.
• Phosphodiesterase-4D phosphorylation and antilipolytic activity are regulated by PAKs.
• Inhibition of PAKs decreases adipogenesis and causes insulin resistance in murine and human adipocytes.
• Activating PAKs in adipocytes may help treat type 2 diabetes by reducing lipolysis and improving insulin sensitivity.

## Graphical abstract

Activation of the FGF1/FGFR1 signaling pathway induces phosphorylation of PDE4D depending on RAFand PAKs. Phosphorylation of PDE4D at S44 is required for inhibition of the cAMP/PKA signaling cascade. Consequently, phosphorylation of HSL at S660 is reduced, resulting in the suppression of lipolysis. In addition to this indirect regulation, PAKs can also directly inhibit HSL activity. Chronic inhibition of PAKs impairs adipogenesis and induces insulin resistance.**PAKs**: p21-activated kinases**PDE4D**: Phosphodiesterase 4DcAMP: cyclicadenosinemonophosphate**PKA**: Protein Kinase A**HSL**: Hormone-sensitive lipase**PI3K**: Phosphoinositide 3-kinase**AKT**: Protein Kinase B**IRS2**: Insulin receptor substrate 2**FGF1:** Fibroblast growth factor 1**INS**: Insulin

**Figure:**
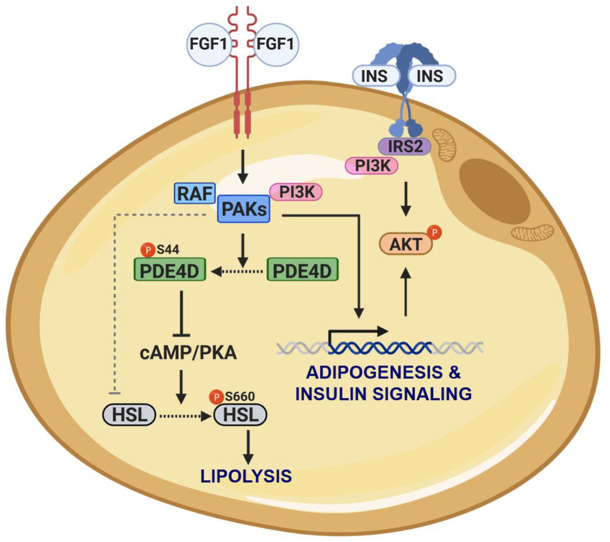

## 1 Introduction

Type 2 diabetes (T2D) is preceded and modified by insulin resistance (IR), a condition in which cells fail to respond effectively to insulin, disrupting glucose and lipid homeostasis. While the development of whole-body IR involves multiple factors, the dysregulation of adipose tissue function is a key contributor. This dysfunction promotes lipotoxicity resulting from elevated circulating lipids and ectopic lipid storage in organs such as the muscle, pancreas, and liver [[1], [2], [3], [4]]. Insulin-resistant adipose tissue is characterized by reduced lipogenic capacity, altered adipokine secretion, chronic inflammation, and heightened basal lipolysis [[5], [6], [7]]. Under normal conditions, insulin suppresses adipose tissue lipolysis to regulate hepatic glucose production (HGP) [8,9]. When adipose tissue lipolysis is reduced, a lower amount of free fatty acids (FFAs) reaches the liver, leading to lower production of hepatic acetyl-CoA. Since acetyl-CoA is a key allosteric activator of pyruvate carboxylase (PC), an essential enzyme for gluconeogenesis, this reduction limits HGP [[8], [9], [10]]. However, the suppression of lipolysis by insulin is impaired in insulin-resistant adipose tissue, resulting in excessive lipolysis, increased HGP, and elevated blood glucose levels [11,12].

Recombinant Fibroblast growth factor 1 (FGF1) injection has been shown to lower blood glucose in glucose-intolerant, insulin-resistant mouse models [13,14]. At the mechanistical level, the glucose-lowering effects of FGF1 are mediated by its suppression of adipose tissue lipolysis through activation of phosphodiesterase 4 (PDE4D) in adipocytes [14,15]. By reducing lipolysis, the FGF1/PDE4D pathway ultimately decreases the flux of gluconeogenic substrates to the liver, lowering HGP. At the molecular level, FGF1-induced phosphorylation of PDE4D at a conserved site (pS44) activates PDE4D, leading to the downregulation of the lipolytic cAMP/PKA pathway and suppression of lipolysis. However, the signaling cascades downstream of FGF1 that lead to suppressing lipolysis and PDE4D activation are unknown. In this study, we investigated the contribution of potential kinases either previously implicated as downstream effectors of FGF1 signaling or those identified by *in silico* prediction algorithms using the amino acid residues surrounding the PDE4D-S44 phosphorylation site. Our study identified RAF and p21-activated Kinases (PAKs) as the modulators of PDE4D phosphorylation and activity. Moreover, chronic inhibition of PAK activity resulted in IR both in mouse and human adipocytes, suggesting a novel role for PAKs in adipocyte metabolism.

## 2 Material and methods

### 2.1 Cell culture

Cell culture experiments were performed using *in vitro* differentiated murine 3T3-L1-preadipocytes (ATCC) and human preadipocytes isolated from subcutaneous white adipose tissue (sc-WAT) (Ethical approval of the University of Tübingen No. 205/2011BO2). All cells were grown at 37 °C in a 5% CO_2_ humid atmosphere. The differentiation of murine preadipocytes was performed according to previously published methods [14]. The human preadipocytes were cultured in alpha-MEM/Ham's F12 (Sigma/Gibco) medium supplemented with 20% FBS (Gibco), l-glutamine (Lonza), chicken embryo extract (Abbexa), and antibiotic-antimycotic (Corning). 2 days after reaching full confluence, the differentiation process was initiated by switching to DMEM/Ham's F12 (Gibco) medium supplemented with 5% FBS, l-glutamine, pantothenic acid (Sigma), biotin (Roth), and antibiotic-antimycotic (growth medium). Throughout the differentiation process, the medium was changed every 2 days. The differentiation started 2 days after reaching full confluence with an 8-day incubation in induction medium based on growth medium with 2 μg/mL apotransferrin (Sigma), 5 μg/mL human insulin (Lilly), 0.5 mM IBMX (Sigma), 1 μM dexamethasone (Sigma), 0.05 mM indomethacin (Sigma) and 1 μM rosiglitazone (Cayman). Subsequently, the cells were cultured for 4 days in differentiation medium, which consisted of induction medium devoid of IBMX and indomethacin. In the final 2-day maturation phase, dexamethasone was omitted from the medium.

### 2.2 Signaling experiments

One day before the experiment, fully differentiated adipocytes were cultured in growth medium without insulin. Before starting the experiment, the cells were incubated for 2 h in a fasting medium consisting of DMEM (Corning) for murine or DMEM/Ham's F12 for human adipocytes with 0.5% BSA (Sigma), 10 mM HEPES (Lonza) and antibiotic-antimycotic. After a 15-minute incubation in KRBH buffer (30 mM HEPES, 120 mM NaCl, 4 mM KH_2_PO_4_, 1 mM MgSO_4_, 0.75 mM CaCl_2_, and 10 mM NaHCO_3_) with 2% fatty acid-free BSA and 5 mM glucose (Roth), the inhibitors were added and incubated for an additional 30 min. Inhibitors used in the study are listed in Supplemental Table 1. Subsequently, either 100 ng/mL Fibroblast Growth Factor 1 (FGF1, Biomol), 100 nM bovine insulin (Sigma), or a vehicle control (KRBH) was added, followed by a 15-minute incubation.

### 2.3 Lipolysis assay

Adipocytes were incubated with inhibitors and FGF1/insulin as described earlier and lipolysis was induced by adding isoproterenol (100 nM for murine adipocytes, 10 nM for human adipocytes, Sigma). The supernatant was collected after 4 h of incubation, and the free fatty acid (FFA) concentrations in the supernatant were measured using the NEFA-HR (2) kit (Wako) and normalized to the volume and protein concentration.

### 2.4 Lipid droplet staining

Adipocytes were incubated for 30 min at 37 °C with KRBH buffer containing 2% BSA, 5 mM glucose, and 200 ng/mL Nile Red dye (Santa Cruz). After 4 washing steps with PBS (Gibco), the cells were incubated in KRBH buffer with 2% BSA and 5 mM glucose and placed in the IncuCyte® S3 Live-Cell Analysis System (Sartorius) to capture images of the cells in green fluorescence (exposure time 300 ms, excitation wavelength 460 nm, emission wavelength 524 nm) and brightfield channel. Images were analysed by IncuCyte® Analysis Software.

### 2.5 Protein extraction, SDS-PAGE and immunoblotting

Adipocytes were lysed in cold RIPA-lysis buffer (50 mM Tris, 150 mM NaCl, 1% NP40, 0.5% NaDoc, 0.1% SDS, 1 mM EDTA, pH 7.5) containing protease- and phosphatase inhibitors (cOmplete and PhosSTOP (Roche)). After ultrasonication in an ice bath for 5 min, samples were centrifuged, and the protein-containing cell lysate was transferred to new reaction tubes and then boiled in Laemmli buffer (BioRad). Pierce™ BCA Protein Assay Kit (Thermo Fisher) was used to determine the protein concentration in the cell lysate. Proteins were separated on 10% SDS-PAGE gels in 1x Tris-Glycine-SDS buffer (BioRad) at 70–120 V and transferred onto nitrocellulose membranes (BioRad) with Trans-Blot® Turbo™ Transfer Buffer (BioRad) at a constant current of 2.5 A and a maximum voltage of 25 V for 20 min using the Trans-Blot Turbo Transfer System (BioRad). The membranes were incubated overnight at 4 °C with the primary antibody, followed by incubation for 2 h with the HRP-conjugated secondary antibody. Antibodies are listed in Supplemental Table 2. The blots were developed with the SuperSignal West Pico PLUS Chemiluminescent Substrate (ThermoFisher Scientific) with Trident femto Western HRP Substrate (GeneTex) and imaged using the BioRad GelDoc System. The Western Blots were quantified using ImageJ.

### 2.6 RNA isolation, cDNA synthesis and qPCR

Adipocytes were lysed using 1 mL of cold QIAzol™ Lysis Reagent (Qiagen). RNA was isolated and purified from the lysates using the NucleoSpin® RNA kit (Macherey–Nagel), following the manufacturer's instructions after a chloroform extraction. The purified RNA was then reverse transcribed into cDNA using the Transcriptor First Strand cDNA Synthesis Kit (Roche), as per the kit protocol, using Mastercycler® nexus Thermocycler (Eppendorf). The synthesized cDNA was amplified and analysed using qPCR on a LightCycler® 480 Instrument II (Roche). The primer sequences used for qPCR are provided in Supplemental Table 3. The housekeeping genes used were *m36B4* for murine and *RSP13* for human samples. The relative expression of the target genes was determined by applying the 2ˆ(-ΔΔCt) formula. The calibrator value was set as the mean ΔCt of DMSO-treated, differentiated adipocyte samples.

### 2.7 PDE4D overexpression by adipose-specific AAV

On Day 7 of differentiation of the 3T3-L1 adipocytes, medium was changed to maturation medium (DMEM 10% FBS supplemented with 5 μg/mL insulin, antibiotic-antimycotic, 10 mM HEPES). Subsequently, either adipose tissue-specific adeno-associated viruses expressing green fluorescent protein (adAAV GFP) or phosphodiesterase 4D (NM_001402885.1; adAAV PDE4D) were added at a concentration of 10ˆ6 genomic copies (GC) per cell as described previously [14]. To enhance transduction efficiency, the AAV was diluted in a solution of PBS with 1 μg/mL Poly-l-Lysine (Sigma). After an incubation period of 48 h, the medium was replaced with fresh maturation medium. The following day, another medium change was performed to culture medium to conduct experiments after an additional 24 h of incubation. adAAVs were produced at the Viral Core Facility of Charité, - Universitätsmedizin Berlin (vcf.charite.de).

### 2.8 cAMP reporter

On day 10 of differentiation, 3T3-L1 adipocytes were washed with PBS containing 1 mM EDTA and detached using 0.05 % Trypsin (Gibco). Cells were resuspended in differentiation medium without antibiotics and antimycotics (DM-), filtered through a 100 μm cell strainer (Greiner Bio-One), counted, and centrifuged for 5 min at 1500 rpm. Cell pellet was resuspended in supplemented SE Cell Line 4D-Nucleofector solution (Lonza). For nucleoporation in nucleovette strips using program CA-133, 0.33 × 10ˆ6 cells in 20 μl volume were mixed with 1 μg of endotoxin-free plasmid. After a 5-minute recovery at room temperature, cells were diluted in DM- and seeded at a density of 0.22 × 10ˆ6 cells/well onto collagen I-coated 8-well chamber slides (Sarstedt). Cells were washed twice with PBS before each medium change. Medium was replaced with growth mediumthe next day, and 48 h post-nucleoporation, cells were fasted for 2–3 h prior to FRET measurement. Cells were imaged in KRBH buffer with 0.1% BSA, pre-treated for 30 min with DMSO or iPAK1-6 at 1 μM. Baseline cAMP levels were monitored for 5 min, followed by addition of 100 nM isoproterenol and observation of the response for 40 min. Imaging was conducted on a Zeiss AXIO Observer Z1 microscope with an automatic XY-Stage (Ludl Electronics), a slide heating system (Idibi) set to 37 °C, a LEDHub high-power LED light engine (Omicron) with a 455 nm LED, and an OptoSplit II emission image splitter (Cairn Research). Pictures were captured with a 20 × /0.5 objective (Zeiss) and a pco.panda 4.2 bi sCMOS camera (Excelitas), using VisiView Software (Visitron Systems) for data collection and image acquisition. The cAMP FRET-Biosensor (YFP-Epac1-CFP) was generously provided by Prof. Viacheslav O. Nikolaev.

### 2.9 cAMP ELISA

Differentiated 3T3-L1 adipocytes were prepared as described in “Signaling Experiments”. After 30 min treatment with DMSO or iPAK1-6 inhibitor (1 μM), isoproterenol was added for 10 min and cells were harvested. cAMP levels were measured by ELISA (cAMP Biotrak™ EIA, Sigma) from the lysates according to the manufacturer's instructions.

### 2.10 Viability assay

3T3-L1 adipocytes or *in vitro* differentiated human adipocytes were treated with DMSO, iPAK1-6 inhibitor (1 μM) or Staurosporin (1 μM) for 2 days. After washing with PBS, the cells were incubated in KRBH buffer with 2% fatty acid-free BSA, 5 mM glucose and 2 μg/mL propidium iodide stain for 5 min. Cells were washed once with PBS and images were taken using Incucyte live cell imaging system (Sartorius) in red fluorescence (exposure time 450 ms, excitation wavelength 585 nm, emission wavelength 635 nm) and brightfield channel. Integrated intensity normalised by the confluence was used for assessing the toxicity of the drugs.

### 2.11 Quantification and statistical analysis

Statistical analyses were conducted using R (version 4.4.1), the figures were created with GraphPad Prism (version 10.1.1). Initially, statistical outliers were identified using the IQR method (1.5 × IQR) and excluded from the analysis. Subsequently, the normal distribution of the data was tested using the Shapiro–Wilk test, and homogeneity of variance was assessed using Levene's test. Based on the results, appropriate statistical tests were selected. For comparing two independent groups, the unpaired Student's t-test or the non-parametric Mann–Whitney U test was used, for comparing multiple independent groups, a one-way ANOVA followed by a Tukey–Kramer post-hoc test, Welch's ANOVA with a subsequent Games-Howell post-hoc test, or the non-parametric Kruskal–Wallis test followed by Dunn's test was performed. All results are presented as means ± SEM for technical replicates. A p-value of <0.05 was considered statistically significant.

## 3 Results

### 3.1 Targeted kinase screening reveals RAF and PAK kinases as regulators of the antilipolytic FGF1/PDE4D signaling pathway

ERK, AKT and mTORC2 were previously identified as signal transducers downstream of FGF1/FGFR1 [16,17]. Inhibition of these kinases, however, did not impair FGF1-mediated suppression of lipolysis (Supp Figure 1A and B). To identify the signaling pathways leading to FGF1/PDE4D-dependent suppression of lipolysis, we next used *in silico* search algorithms to identify potential kinases that phosphorylate the PDE4D-S44 site [18,19]. The prediction algorithm suggested p21-associated kinase 4 (PAK4) as top candidate, together with other PAKs (PAK1 to PAK6) in the top hit list (Supp Fig. 1C). We conducted a targeted screen in 3T3-L1 adipocytes to evaluate the role of the kinases listed, focusing on those with available specific inhibitors, along with other potential downstream kinases of FGF1/FGFR1, in mediating the suppression of lipolysis by FGF1. Among the inhibitors we tested, only the pan-RAF inhibitor (LY3009120) and pan-PAK inhibitor (PF-37580937 - iPAK1-6) impaired FGF1-induced suppression of lipolysis (Supp Fig. 1D). In contrast, inhibition of S6K1, Cdc42, RAC, CaMKII, or MSK did not reverse suppression of lipolysis by FGF1 while inhibition of AURORA, RSK, MEK, or MNK exhibited only mild effects on lipolysis (Supp Fig. 1D).

**Figure 1:**
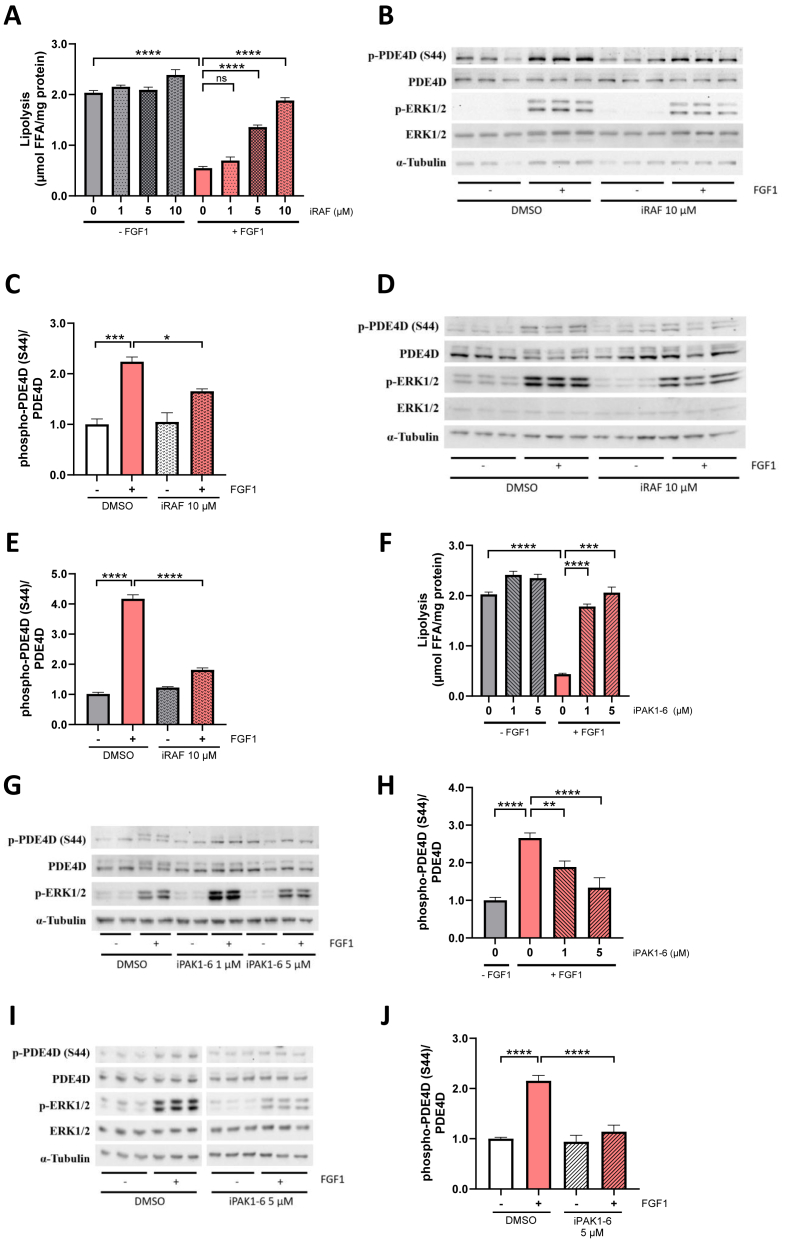
**Targeted kinase screening reveals RAF-PAK kinases as regulators of PDE4D-S44 phosphorylation.** **(A)** Quantification of lipolysis in 3T3-L1 adipocytes after treatment with increasing concentrations of 1, 5 and 10 μM of RAF inhibitor LY3009120 and stimulation with KRBH or FGF1 (100 ng/mL) and isoproterenol (100 nM) (n = 5–6 per inhibitor dose, n = 11–12 for DMSO). **(B)** Western Blots showing PDE4D-S44 phosphorylation in cells treated with 10 μM RAF inhibitor and stimulated with KRBH or FGF1. **(C)** Quantification of PDE4D-S44 phosphorylation normalised to total PDE4D levels (n = 3). **(D)** Western Blots showing PDE4D-S44 phosphorylation in cells treated with 10 μM RAF inhibitor and stimulated with KRBH or FGF1 and isoproterenol. **(E)** Quantification of PDE4D-S44 phosphorylation normalised to total PDE4D levels (n = 3). **(F)** Lipolysis in 3T3-L1 adipocytes after treatment with DMSO (n = 21) and PAK1-6 inhibitor PF-3758309 at 1 μM (n = 15) or 5 μM (n = 6) and stimulation with KRBH or FGF1 and isoproterenol. **(G)** Western Blot showing PDE4D-S44 phosphorylation after treatment with DMSO or 1 and 5 μM PAK1-6 inhibitor and stimulation with KRBH or FGF1 and isoproterenol. **(H)** Quantification of PDE4D-S44 phosphorylation normalised to total PDE4D levels (n = 4–8). **(I)** Western Blot showing PDE4D-S44 phosphorylation after treatment with DMSO, PAK1-6 inhibitor at 1 μM or 5 μM and stimulation with KRBH or FGF1. **(J)** Quantification of PDE4D-S44 phosphorylation normalised to total PDE4D levels (n = 7). Data are presented as mean ± SEM of technical replicates (∗p < 0.05, ∗∗p < 0.01, ∗∗∗p < 0.001, ∗∗∗∗p < 0.0001, ns not significant).

RAF serves as upstream activator of MAPK signaling, a pathway that is induced by FGF1/FGFR1 axis [20]. Inhibition of RAF in 3T3-L1 adipocytes decreased FGF1-dependent suppression of lipolysis in a dose-dependent manner (Figure 1A). Since phosphorylation and activation of PDE4D are required for FGF1-mediated suppression of lipolysis, we next tested the effect of RAF inhibition on PDE4D-S44 phosphorylation. Inhibition of RAF decreased the FGF1-induced PDE4D phosphorylation both under normal (Figure 1B,C) and lipolytic (i.e. isoproterenol-treated) conditions (Figure 1D,E), indicating that the RAF signaling arm of FGF1/FGFR1 is required for FGF1-induced PDE4D phosphorylation and suppression of lipolysis. In addition to RAF, our screen revealed that PAK signaling plays a role in mediating the antilipolytic effects of the FGF1/PDE4D pathway. Inhibition of PAK prevented the FGF1 effect on lipolysis in a dose-dependent manner (Figure 1F). Moreover, FGF1-induced hyperphosphorylation of PDE4D was inhibited by iPAK1-6 in parallel to its effect on lipolysis (Figure 1G,H). A similar effect on PDE4D phosphorylation was observed after FGF1 stimulation under non-lipolytic conditions (Figure 1I,J). Together, our data indicate that FGF1-induced phosphorylation of PDE4D and suppression of lipolysis are dependent on RAF and PAK signaling pathways.

### 3.2 Inhibition of Group I PAKs is not sufficient to prevent FGF1-induced phosphorylation of PDE4D and suppression of lipolysis

**Figure 2:**
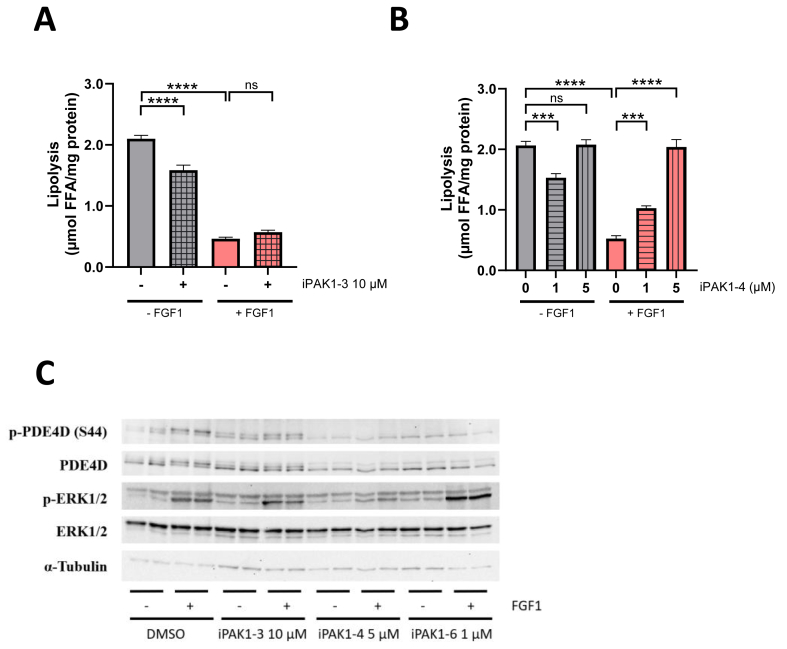
**Inhibition of Group I PAKs is not sufficient to prevent FGF1-induced phosphorylation of PDE4D and suppression of lipolysis.** Quantification of lipolysis in 3T3-L1 adipocytes after stimulation with KRBH or FGF1 (100 ng/mL) and isoproterenol (100 nM) after treatment with **(A)** PAK1-3 inhibitor FRAX597 at 10 μM (n = 16–19) or **(B)** PAK1-4 inhibitor FRAX486 at 1 μM and 5 μM (n = 11 for DMSO, n = 4–5 for iPAK1-4 at 1 μM, n = 6 for iPAK1-4 at 5 μM). **(C)** Western Blot showing PDE4D-S44 phosphorylation after treatment with DMSO, PAK1-3 inhibitor (10 μM), PAK1-4 inhibitor (5 μM) or PAK1-6 inhibitor (1 μM) and stimulation with KRBH or FGF1 and isoproterenol. Data are presented as mean ± SEM of technical replicates (∗∗∗p < 0.001, ∗∗∗∗p < 0.0001, ns not significant).

The role of PAKs in cellular growth has been extensively studied, leading to the development of several inhibitors targeting sub-groups of PAK enzymes [21] i.e. class I (PAK1 to PAK3) and class II (PAK4 to PAK6) kinases [22]. PF-3758309 was developed as a potent PAK4 inhibitor but also exhibits inhibitory activity against other PAK family members, making it a broad-spectrum PAK inhibitor [23]. To determine which PAK isoforms are involved in FGF1-mediated suppression of lipolysis, we systematically employed several PAK inhibitors in 3T3-L1 adipocytes. Inhibition of PAK1 and PAK2 (by NVS-PAK1-1) [24] did not affect FGF1-mediated suppression of lipolysis and PDE4D hyperphosphorylation (Supp Figs. 2A and 2B). Moreover, inhibition of PAK1 to PAK3 (by FRAX597) [25] had no impact on the suppression of lipolysis by FGF1 whereas inhibition of PAK1 to PAK4 (by FRAX486) [26] prevented the suppression of lipolysis by FGF1 (Figure 2A,B). In agreement with its effect on lipolysis, the inhibition of PAK1-4 prevented FGF1-induced PDE4D hyperphosphorylation similar to the inhibition of PAK1-6 (Figure 2C). Our data indicate that while inhibition of class I PAKs is not sufficient to prevent FGF1/PDE4D-induced suppression of lipolysis, inhibiting PAK4 in addition to PAK1 to PAK3 abrogates both PDE4D phosphorylation and suppression of lipolysis by FGF1.

### 3.3 Suppression of cAMP/PKA signaling by the FGF1/PDE4D pathway is mediated by PAKs

**Figure 3:**
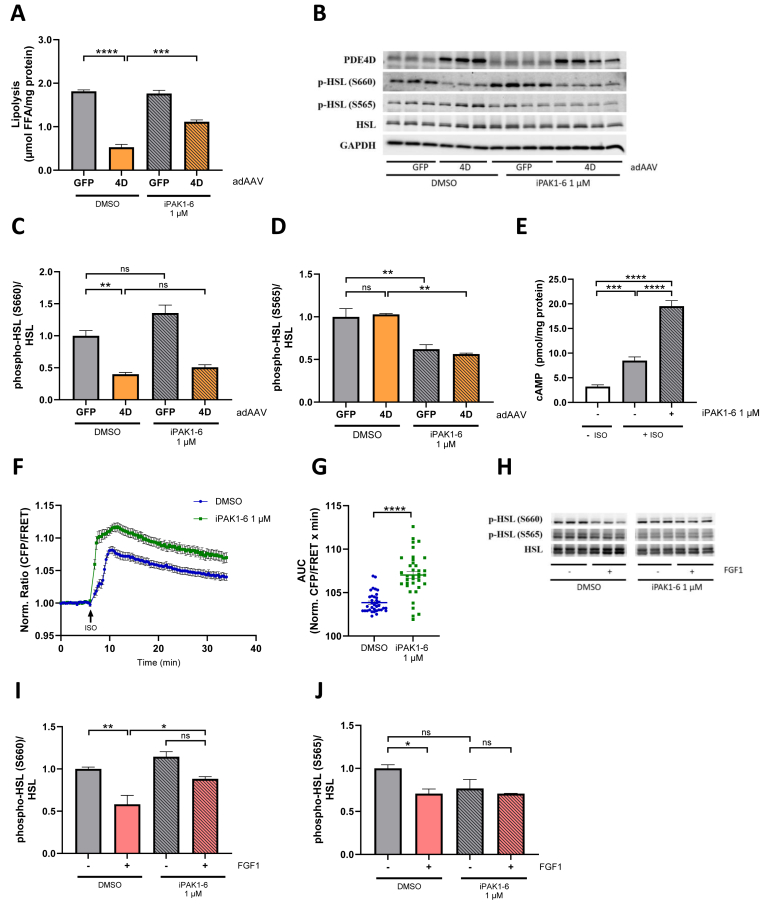
**Suppression of cAMP/PKA signaling by the FGF1/PDE4D pathway is mediated by PAKs.** **(A)** Quantification of lipolysis in 3T3-L1 adipocytes infected with an adipose tissue-specific adeno-associated virus (adAAV) expressing GFP or PDE4D. Cells were treated with DMSO or PAK1-6 inhibitor (1 μM) before induction of lipolysis with isoproterenol (100 nM) (n = 3–4). **(B)** Western Blot showing HSL-S660 and HSL-S565 phosphorylation after treatment with PAK1-6 inhibitor (1 μM) and stimulation with isoproterenol in cells overexpressing PDE4D. Quantification of HSL-S660 phosphorylation **(C)** and HSL-S565 phosphorylation **(D)** normalised to total HSL levels. **(E)** cAMP levels in 3T3-L1 adipocytes measured 10 min after treatment with 100 nM isoproterenol (ISO), following pretreatment with either DMSO or 1 μM PAK1-6 inhibitor for 30 min prior to ISO addition. A condition without drug treatment was included as the baseline for cAMP levels (n = 8) **(F)** Normalised cAMP levels in 3T3-L1 adipocytes measured with a FRET-based cAMP reporter with iPAK1-6 (1 μM) or without (DMSO) after 30 min pre-treatment. Time of ISO addition is indicated with a black arrow. **(G)** Area under the curve (AUC) of the traces shown in **(F)** for individual cells (n = 37–38) **(H)** Western Blot showing HSL-S660 and HSL-S565 phosphorylation after treatment with DMSO or PAK1-6 inhibitor (1 μM) and stimulation with KRBH or FGF1 and isoproterenol. **(I)** Quantification of HSL-S660 phosphorylation normalised to total HSL levels (n = 3) **(J)** Quantification of HSL-S565 phosphorylation normalised to total HSL levels (n = 3). Data are presented as mean ± SEM of technical replicates (∗p < 0.05, ∗∗p < 0.01, ∗∗∗p < 0.001, ∗∗∗∗p < 0.0001, ns not significant).

Overexpression of PDE4D, but not a S44A mutant PDE4D, inhibits lipolysis in adipocytes [14]. Assuming PAKs indeed regulate PDE4D phosphorylation through S44, we hypothesized that inhibition of PAK activity may impair the ability of PDE4D-overexpression to suppress lipolysis. Consistent with this hypothesis, inhibition of PAKs employing iPAK1-6 attenuated the repression of lipolysis by PDE4D overexpression in 3T3-L1 adipocytes (Figure 3A and Supp Fig. 3A). Overexpression of PDE4D decreases phosphorylation of hormone-sensitive lipase (HSL) at S660, a site required for activation of HSL by protein kinase A (PKA) [14,27] (Figure 3B). We observed that inhibition of PAKs resulted in slightly higher pHSL-S660 levels in control (green fluorescent protein, GFP) or after PDE4D overexpression, albeit no statistically significant differences were observed between PDE4D overexpressing adipocytes that are treated with vehicle or iPAK1-6 (Figure 3C). A recent study has identified PAK4 as potentially responsible for phosphorylating the HSL-S565 site, previously recognised as an inhibitory site [28,29]. Overexpression of PDE4D did not change the phosphorylation status of HSL at S565, however, inhibition of PAKs decreased pHSL-S565 levels regardless of the GFP or PDE4D overexpression (Figure 3B, D). Next, we examined whether PAK inhibition would alter the cells' cAMP levels which would agree with the proposed effects on PDE4D phosphorylation and activity. Indeed, we observed increased total cAMP levels upon PAK inhibition after lipolytic stimulation with isoproterenol (ISO) (Figure 3E). Moreover, we measured dynamic cAMP response by using a FRET-based cAMP-biosensor [30] showing a significantly enhanced cAMP response following PAK inhibition (Figure 3F, G). Consequently, the FGF1 effect on suppressing pHSL-S660 phosphorylation was impaired upon PAK inhibition (Figure 3H, I). This effect was also observed when iPAK1-4 or iRAF was used, confirming their impact on lipolysis and PDE4D phosphorylation (Supp Fig. 3B). FGF1 treatment reduced pHSL-S565 levels, but this effect was abolished by iPAK1-6 treatment. (Figure 3H, J).

To differentiate whether PAKs inhibit lipolysis directly by regulating HSL phosphorylation, as suggested by a recent study [28], or indirectly via activation of PDE4D, we tested whether insulin could still suppress lipolysis in the presence of a PAK inhibitor. If PAK inhibition directly reduces pHSL-S565 and increases pHSL-S660, as proposed [28], it would be expected to impair insulin's antilipolytic effect, as insulin acts through PDE3B to lower cAMP levels and thereby reducing pHSL-S660 phosphorylation. However, inhibition of PAK activity with iPAK1-6 did not compromise insulin-mediated suppression of lipolysis (Supplementary Fig. 3C). Our findings indicate that RAF and PAK signaling pathways mediate the FGF1-induced PDE4D phosphorylation to subsequently repress lipolysis. PAK regulates lipolysis primarily through modulation of a PDE4D-sensitive cAMP/PKA pathway, rather than through direct phosphorylation of HSL.

### 3.4 Inhibition of PAKs decreases adipogenesis and causes IR in murine and human adipocytes

**Figure 4:**
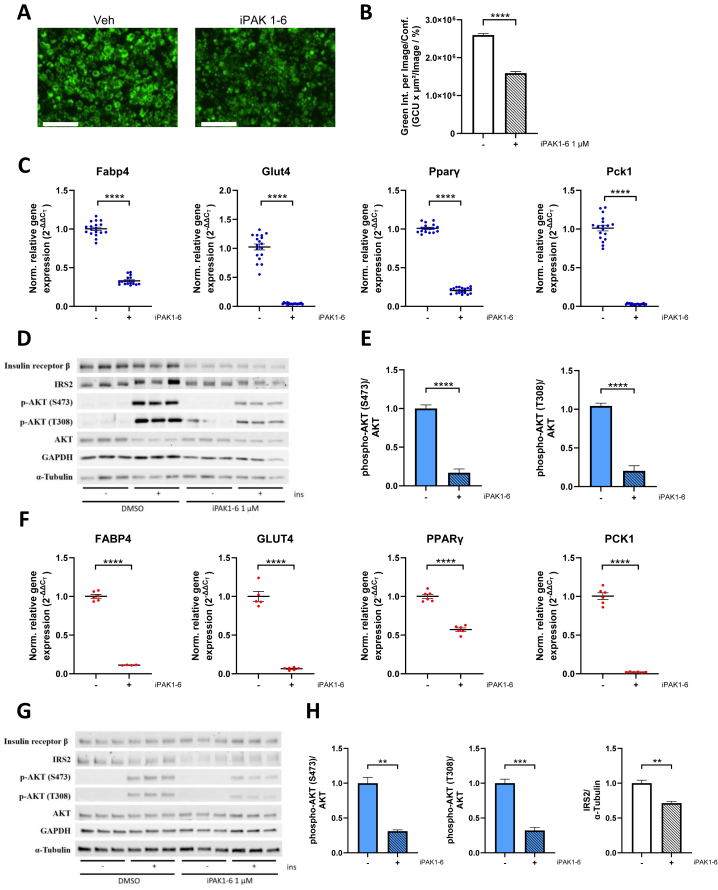
**Inhibition of PAKs decreases adipogenesis and causes IR in murine and human adipocytes.** **(A)** Fluorescent images of 3T3-L1 adipocytes at day 10 of differentiation after 2 days of treatment with DMSO or PAK1-6 inhibitor (1 μM) after staining with Nile Red. Scale bar indicates 200 μM. **(B)** Quantification of the green integrated intensity normalised to phase area confluence (n = 8–10). **(C)** Relative gene expression levels of *Fabp4*, *Glut4*, *Pparγ*, and *Pck1* after 2-day treatment with DMSO or PAK1-6 inhibitor (1 μM) from day 8 to day 10 of differentiation. Cells were lysed on day 10, and gene expression levels were quantified by qRT-PCR and normalised to *m36B4* (n = 16–18). **(D)** Western Blots of 3T3-L1 adipocytes after 2-day treatment with DMSO or PAK1-6 inhibitor (1 μM) and stimulation with KRBH or insulin (10 nM) on day 11 and **(E)** Quantification of phosphorylation of AKT at S473 or T308 normalised to total AKT (n = 5–6). **(F)** Relative gene expression levels of *FABP4*, *GLUT4*, *PPARγ* and *PCK1* after treatment of human subcutaneous adipocytes with DMSO or PAK1-6 inhibitor (1 μM) from day 15 to day 17 of differentiation. The cells were lysed on day 17, and gene expression levels were quantified by qRT-PCR and normalised to *RPS13* (n = 4–6). **(G)** Western Blots and **(H)** quantification of phosphorylation of AKT at S473 or T308 normalised to total AKT levels and IRS2 normalised to α-tubulin in human subcutaneous adipocytes after 2-day treatment with DMSO or PAK1-6 inhibitor (1 μM) from day 14 to day 16 of differentiation and stimulation with KRBH or insulin (1 nM) on day 17 (n = 3–6). Data are presented as mean ± SEM of technical replicates (∗∗p < 0.01, ∗∗∗p < 0.001, ∗∗∗∗p < 0.0001). (For interpretation of the references to color in this figure legend, the reader is referred to the Web version of this article.)

Upon observing the involvement of PAK signaling in lipolysis and cAMP regulation in an acute setting, we tested whether chronic inhibition of PAKs affects adipogenesis and insulin signaling in murine adipocytes. Interestingly, 2 days of PAK1-6 inhibition during differentiation decreased lipid accumulation in adipocytes (Figure 4A, B). Accordingly, gene expression analysis of adipogenic markers such as *Fabp4*, *Glut4*, *Pparγ*, *Pck1*, *AdipoQ*, and *Lipe* revealed a significant downregulation upon inhibition of PAKs (Figure 4C and Supp Fig. 4A). In addition, *insulin receptor* (*InsR*) and *Irs2* transcript levels were also downregulated, indicating a potential impairment of insulin signaling. Of note, *Irs1* mRNA levels were elevated upon PAK inhibition, possibly as a compensatory mechanism for decreased *InsR* and *Irs2* (Supp Fig. 4A). We next tested whether the downregulation of *InsR* and *Irs2* at RNA levels also affected the corresponding proteins and insulin signaling. We observed decreased insulin receptor β and IRS2 protein levels upon PAK inhibition and reduced insulin signaling as assessed by insulin-stimulated AKT phosphorylation at S473 and T308 (Figure 4D,E, Supp Fig. 4B). To rule out the possibility that chronic PAK inhibition causes cytotoxicity, we quantified cell death and did not observe major differences between iPAK1-6 treated and untreated 3T3-L1 mouse adipocytes (Supp Fig. 4C).

Since PAK inhibition enhances lipolysis and may thereby contribute to IR, we investigated whether stimulating lipolysis through PDE inhibition would similarly impair insulin sensitivity. However, treatment with either the non-specific PDE inhibitor IBMX or a combination of PDE3 and PDE4 inhibitors for 2 days did not induce IR as assessed by insulin-induced AKT phosphorylation (Supp Fig. 4D). These findings suggest that, at least *in vitro*, the IR caused by PAK inhibition is primarily driven by altered regulation of adipogenic genes rather than increased lipolysis alone.

To test how these findings potentially translate into human adipocyte metabolism, we next examined whether PAK activity is similarly required for FGF1/PDE4D-dependent suppression of lipolysis in human adipocyte cultures. Acute inhibition of PAK activity prior to the lipolysis assay abolished the suppressive effect of FGF1 on lipolysis in human adipocytes. Furthermore, PAK inhibition potentiated isoproterenol-stimulated lipolysis, consistent with the observations in 3T3-L1 adipocytes (Supp Fig. 4E).

While we observed a conserved acute effect of PAK inhibition in human adipocytes, we tested whether chronic PAK inhibition during differentiation impairs adipocyte differentiation and insulin signaling in primary human adipocyte cultures. For this, human preadipocytes isolated from subcutaneous adipose tissue were differentiated *in vitro* and treated with iPAK1-6 for 2 days. The adipogenic markers *FABP4, GLUT4, PCK1, PPARγ, ADIPOQ, CD36* and *LIPE* were downregulated in human adipocytes upon PAK inhibition (Figure 4F and Supp Fig. 4F). The gene expression levels of *IRS1* were also downregulated while *INSR* and *IRS2* were upregulated upon iPAK1-6 treatment (Supp Fig. 4F). Similar to what we observed in murine adipocytes, PAK inhibition resulted in decreased insulin signaling as assessed by insulin-stimulated AKT phosphorylation at S473 and T308 (Figure 4G, H). Lower IRS2 protein levels in iPAK-treated adipocytes could, in part contribute to lower insulin signaling (Figure 4G) [31]. While we observed lower InsR protein levels in mouse adipocytes, there was no observable difference in human adipocytes between treatment groups, probably due to increased INSR RNA levels in human adipocytes (Figure 4G and Supp Fig. 4F). 2-day iPAK1-6 treatment did not induce cell death in human adipocytes excluding the non-specific effect of PAK inhibition on adipogenesis or insulin signaling (Supp Fig. 4G).

## 4 Discussion

Until now, PI3K was the only known kinase mediating the antilipolytic function of FGF1 by relaying the signaling cascade to PDE4D phosphorylation. In this study, we uncovered RAF and PAK as novel regulators of lipolysis within the FGF1/PDE4D axis. While PI3K/AKT and RAS/RAF/MAPK pathways are canonical pathways for growth factor signaling [32,33], neither AKT or mTOR nor ERK inhibition prevented the antilipolytic role of the FGF1/PDE4D pathway indicating a novel signaling cascade regulating lipolysis via FGF1/PDE4D. Previous studies showed that activation of PI3K can lead to RAS-RAF activation, and inhibition of PI3K activity by wortmannin interfered with the RAS-RAF pathway [34]. Moreover, the interaction of RAF and PAK proteins results in their concomitant activation [[35], [36], [37], [38]]. Hence, we hypothesize the PI3K-RAF/PAK pathway as the major signaling arm of FGF1 resulting in PDE4D phosphorylation and suppression of lipolysis. Our data indicate that inhibition of PAK1 to PAK3 alone was insufficient to prevent the antilipolytic function of the FGF1/PDE4D pathway showing the involvement of class II PAKs. In accordance with this data, recently PAK4 was suggested to be involved in the regulation of lipolysis supporting the antilipolytic role of class II PAKs [28]. The authors found that PAK4 protein levels are regulated by fasting, potentially controlled by PKA-induced degradation. They suggested that PAK4 suppresses lipolysis by phosphorylating HSL at S565 and FABP4 at T126. While our data are consistent with a possible antilipolytic action of PAK4, another mechanistic explanation is the negative regulation of the cAMP/PKA pathway by PAK via prevention of phosphorylation of HSL at S660 which is a critical site for activation of lipolysis in adipocytes [39]. FGF1 treatment reduced phosphorylation of HSL at both S565 and S660 residues indicating that decreased pHSL-S565 levels do not necessarily cause higher lipolysis. In line with this hypothesis, we observed the prevention of PDE4D activation, increased cAMP levels, and blunting of the FGF1 antilipolytic function and its effect on HSL-S660 phosphorylation upon PAK inhibition. In contrast, Yu et al. did not address the potential effect of PAK4 inhibition or deletion on the cAMP/PKA pathway in adipocytes. Moreover, we show that inhibition of the PAK activity does not abrogate the antilipolytic function of insulin. This data highlights that the PAKs act mainly through PDE4D, not by directly affecting the HSL phosphorylation status. If PAK inhibition directly decreases pHSL-S565 and increases pHSL-S660, as proposed in Yu et al. antilipolytic effects of insulin, which rely on a PDE3B-mediated depletion of cAMP to suppress pHSL-S660 phosphorylation, should also be impaired. Although Yu et al. provide evidence for a partial protection from HFD-induced metabolic perturbations when PAK4 was inhibited in mice. However, inhibitor treatment started at the beginning of HFD feeding, and there was a substantial weight difference between the mice groups (control vs PAK4 inhibitor) when metabolic characterization was performed. Thus, it would be of interest to see the effect of PAK inhibitor treatment on glucose homeostasis when mice have developed HFD feeding-associated IR, as such a setup would better simulate the therapeutic potential. We previously showed that inhibition of lipolysis by adipose-specific overexpression of PDE4D decreased plasma glucose concentrations and ameliorated glucose tolerance in HFD-fed or *ob/ob* mice [14]. Hence, supported by our previous data and the data presented herein, we believe that the suppression of lipolysis by PAK via pharmacological activation and/or overexpression in adipocytes could be beneficial for the regulation of glucose homeostasis in metabolic disease.

In addition to its acute role in the regulation of lipolysis by FGF1/PDE4D, PAK inhibition for 2 days during differentiation impaired adipogenesis and decreased insulin sensitivity in both murine and human adipocytes. It is well established that adipogenesis markers such as *Pparγ, Fabp4, Pck1* and *AdipoQ* expression levels correlate closely with insulin sensitivity in adipocytes [[40], [41], [42], [43]]. We observed the downregulation of these genes upon PAK inhibition. Moreover, lower IRS2 protein levels were observed in both mouse and human adipocytes, which could contribute to impaired insulin signaling. While induction of lipolysis over two days using PDE inhibition—either with IBMX or a combination of PDE3 and PDE4 inhibitors—did not lead to significant insulin resistance, our data suggest that PAK inhibition modulates insulin signaling mainly independently of its role in regulating lipolysis. It would be of interest to investigate in the future the direct effect of PAKs on posttranslational regulation of transcription factors involved in adipogenesis and insulin signaling. To our knowledge for the first time, we demonstrate the involvement of PAK signaling in adipogenesis and IR in adipocytes. Further studies are required to investigate the contribution of each individual PAK (1–6) on cAMP/PKA dynamics regulated by FGF1/PDE4D. We believe that answers to this current gap in knowledge will fundamentally change our understanding of the regulation of lipolysis, insulin signaling, and glucose metabolism.

## CRediT authorship contribution statement

**Judith Seigner:** Writing – review & editing, Visualization, Methodology, Investigation, Formal analysis, Data curation, Conceptualization. **Johannes Krier:** Writing – review & editing, Methodology, Investigation, Formal analysis. **David Spähn:** Writing – review & editing, Methodology, Formal analysis. **Leontine Sandforth:** Writing – review & editing, Methodology. **Judith L. Nono:** Methodology. **Robert Lukowski:** Writing – review & editing, Supervision, Resources, Methodology. **Andreas L. Birkenfeld:** Writing – review & editing, Supervision, Resources, Funding acquisition. **Gencer Sancar:** Writing – original draft, Supervision, Resources, Project administration, Methodology, Investigation, Funding acquisition, Conceptualization.

## Funding

This work is supported by the German Center for Diabetes Research (DZD), Neuherberg-Munich, Germany, and by grants from the German Research Foundation (DFG) (RTG2816/1 to ALB, and DFG Project number 562635405 to GS). Work in the Lukowski laboratory was funded by the German Research Foundation (DFG) via individual grant LU 1490/10-1. RL and DS are members/associates of the GRK2381: “cGMP: From Bedside to Bench”, DFG grant number 335549539.

## Declaration of competing interest

The authors declare that they have no known competing financial interests or personal relationships that could have appeared to influence the work reported in this paper.

## Data Availability

Data will be made available on request.
