# Supplementary material for: p21-activated kinases (PAKs) regulate FGF1/PDE4D antilipolytic pathway and insulin resistance in adipocytes
Source: Mol Metab. 2025 Jul 12;99:102210. doi: 10.1016/j.molmet.2025.102210 (PMC12311537; doi:10.1016/j.molmet.2025.102210)
Supplement: Multimedia component 1 [file mmc1.docx]

**Supplemental Tables**

**Supplemental Table1**. **Table showing the inhibitors used in the study**

| **Inhibitor** | **Origin** | **Catalog #** |
| --- | --- | --- |
| AZD-8055 | Focus Biomolecules | 10-4816 |
| Cilostamide | Selleck Chemicals | S5806 |
| eFT508 | Cayman Chemical | 21957 |
| EHT 1864 | Cayman Chemical | 17258 |
| FRAX486 | MedChemExpress | HY-15542B |
| FRAX597 | MedChemExpress | HY-15542A |
| IBMX | Sigma Aldrich | I5879 |
| KN-93 | Selleck Chemicals | S6787 |
| LJH685 | Selleck Chemicals | S7870 |
| LY3009120 | Sigma Aldrich | SML3055 |
| Mirdametinib, PD0325901 | Selleck Chemicals | HY-10254 |
| MK-2206 monohydro-chlorid | LKT Laboratories Inc | M4000 |
| ML141 | Selleck Chemicals | S7686 |
| NVS-1-1 | Medchem express | HY-100519 |
| PF-3758309 dihydrochloride | Tocris Bioscience | 6005 |
| PF-4708671 | Sigma Aldrich | PZ0143 |
| Ravoxertinib, GDC-0994 | Medchem express | HY-15947 |
| Roflumilast | Selleck Chemicals | S2131 |
| SB747651A dihydrochloride | Tocris Bioscience | 4630 |
| SCH772984 | Selleck Chemicals | S7101 |
| Tozasertib | SantaCruz Biotechnology | Sc-358750 |

**Supplemental Table 2**. **Table showing the antibodies used in the study.**

| Antibody | Manufacturer | dilution | Catalog # |
| --- | --- | --- | --- |
| α-Tubulin (DM1A) | Merck Millipore | 1:2.000 | CP06 |
| Akt (pan) (40D4) | Cell Signaling | 1:1.000 | 2920 |
| GAPDH | Invitrogen | 1:1.000 | MA5-15738 |
| Goat anti Mouse IgG (H/L):HRP | Bio-Rad Laboratories, Inc. | 1:5.000 | 5178-2504 |
| Goat Anti-Rabbit IgG (H+L)-HRP Conjugate | Bio-Rad Laboratories, Inc. | 1:5.000 | 1706515 |
| HSL | Cell Signaling | 1:1.000 | 4107 |
| Insulin receptor β | Cell Signaling | 1:1.000 | 3020 |
| IRS2 | Cell Signaling | 1:1.000 | 3089 |
| p44/42 MAPK (ERK1/2) | Cell Signaling | 1:2.000 | 9102 |
| PDE4D | Proteintech | 1:1.000 | 12918-1-AP |
| PDE4D-pS44 | See References [14; 44] | 1:500 | NA |
| Phospho-AKT (S473) | Cell Signaling | 1:1.000 | 9271 |
| Phospho-AKT (T308) | Cell Signaling | 1:1.000 | 13038 |
| Phospho-HSL (S565) | Cell Signaling | 1:1.000 | 4137 |
| Phospho-HSL (S660) | Cell Signaling | 1:1.000 | 45804 |
| Phospho-p44/42 MAPK (ERK1/2)  (T202/Y204, T185/Y187) | Cell Signaling | 1:1.000 | 9101 |

**Supplemental Table 3**. **Table showing the primers used in the study for genes expression analysis.**

| Gene | Forward sequence | Reverse sequence |
| --- | --- | --- |
| AdipoQ | GAGGGAGAAAAGGCGAGCTT | GGGTTCCTCTGCTTGTTCCA |
| ADIPOQ | GGTGAGAAGGGTGAGAAAGGA | TTTCACCGATGTCTCCCTTAG |
| Cd36 | GGACATTGAGATTCTTTTCCTCTG | GCAAAGGCATTGGCTGGAAGAAC |
| CD36 | CAGGTCAACCTATTGGTCAAGCC | GCCTTCTCATCACCAATGGTCC |
| Fabp4 | AAGAAGTGGGAGTGGGCTTT | CTGTCGTCTGCGGTGATTT |
| FABP4 | CCTTTAAAAATACTGAGATTTCCTTCA | GGACACCCCCATCTAAGGTT |
| Glut4 | CTATGCTGGCCAACAATGTC | CCCTGATGTTAGCCCTGAGT |
| GLUT4 | CTGTGCCATCCTGATGACTG | CGTAGCTCATGGCTGGAACT |
| InsR | AGATGAGAGGTGCAGTGTGGCT | GGTTCCTTTGGCTCTTGCCACA |
| INSR | GCAACATCACCCACTACCTGGT | GAATGGTGGAGACCAGGTCCTC |
| Irs1 | TGTCACCCAGTGGTAGTTGCTC | CTCTCAACAGGAGGTTTGGCATG |
| IRS1 | AGTCTGTCGTCCAGTAGCACCA | ACTGGAGCCATACTCATCCGAG |
| Irs2 | CCAGTAAACGGAGGTGGCTACA | CCATAGACAGCTTGGAGCCACA |
| IRS2 | CCTGCCCCCTGCCAACACCT | TATGACATCCTGGTGATAAAGCC |
| Lipe | GATTTACGCACGATGACACAGT | ACCTGCAAAGACATTAGACAGC |
| LIPE | AGCCTTCTGGAACATCACCGAG | TCGGCAGTCAGTGGCATCTCAA |
| m36B4 | AGATTCGGGATATGCTGTTGGC | TCGGGTCCTAGACCAGTGTTC |
| Pck1 | CCACAGCTGCTGCAGAACA | GAAGGGTCGCATGGCAAA |
| PCK1 | ATTGATGCTGCCTGGGAGT | CCCCACAAAGACTCCATGTT |
| Pparγ | TCGCTGATGCACTGCCTATG | GAGAGGTCCACAGAGCTGATT |
| PPARγ | TTGCTGTCATTATTCTCAGTGGA | GAGGACTCAGGGTGGTTCAG |
| RPS13 | CCCCACTTGGTTGAAGTTGA | ACACCATGTGAATCTCTCAGGA |
