# Supplementary material for: p21-activated kinases (PAKs) regulate FGF1/PDE4D antilipolytic pathway and insulin resistance in adipocytes
Source: Mol Metab. 2025 Jul 12;99:102210. doi: 10.1016/j.molmet.2025.102210 (PMC12311537; doi:10.1016/j.molmet.2025.102210)
Supplement: Multimedia component 2 [file mmc2.pdf]

A

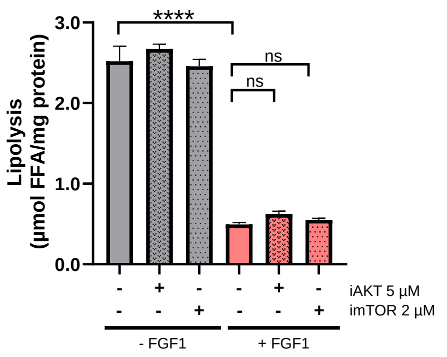

B

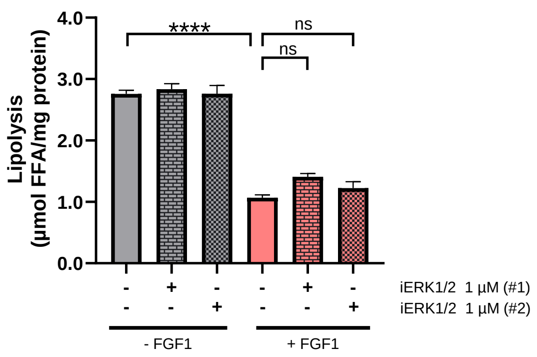

C

Rank	Kinase	Kinase Group	Score	Log2-Score	Site Percentile
**1**	**PAK4**	**STE**	**187,438**	**7,550**	**99,90**
2	CAMK1A	CAMK	150,509	7,234	99,91
3	PKG1	AGC	144,677	7,177	99,86
**4**	**PAK5**	**STE**	**117,995**	**6,883**	**99,84**
5	DAPK1	CAMK	62,572	5,967	99,72
6	MSK2	AGC	59,821	5,903	99,62
**7**	**PAK2**	**STE**	**58,626**	**5,873**	**99,87**
8	CAMK1D	CAMK	57,713	5,851	99,57
9	PKACA	AGC	55,232	5,787	98,69
10	MYLK4	CAMK	53,123	5,731	99,58
11	AKT1	AGC	49,309	5,624	99,29
12	CAMK1G	CAMK	47,255	5,562	99,74
**13**	**PAK1**	**STE**	**45,497**	**5,508**	**99,63**
**14**	**PAK3**	**STE**	**44,913**	**5,489**	**99,76**
15	PKG2	AGC	42,263	5,401	99,27
16	SGK1	AGC	40,980	5,357	98,36
17	CAMK4	CAMK	40,740	5,348	99,88
18	AKT3	AGC	40,439	5,338	98,85
19	MSK1	AGC	38,815	5,279	98,83
20	AURA	Other	37,054	5,212	99,06
21	AKT2	AGC	34,953	5,127	98,76
22	MRCKA	AGC	34,855	5,123	99,31
23	DYRK1A	CMGC	34,383	5,104	97,38
24	CHK2	CAMK	33,242	5,055	99,29
25	MRCKB	AGC	32,829	5,037	99,21
**26**	**PAK6**	**STE**	**29,591**	**4,887**	**99,15**
27	RSK3	AGC	28,982	4,857	98,93
28	P70S6K	AGC	28,712	4,844	98,89
29	skMLCK	CAMK	26,707	4,739	99,63
30	MAPKAPK5	CAMK	25,434	4,669	99,66
**100**	**RAF1**	**TKL**	**38,481**	**1,944**	**98,73**

D

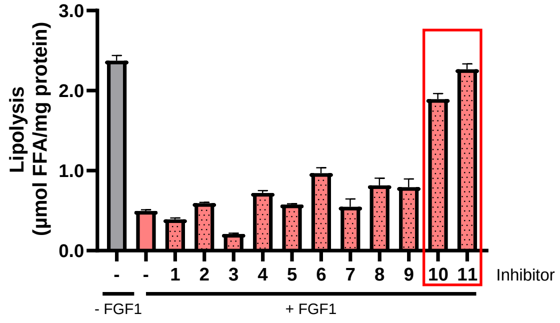

Supplemental Figure 1

A

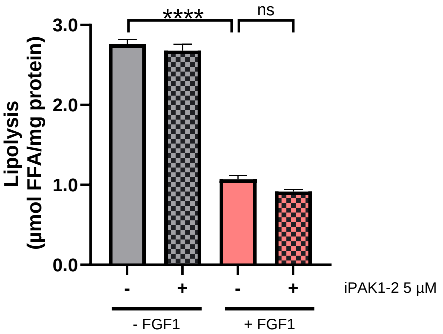

B

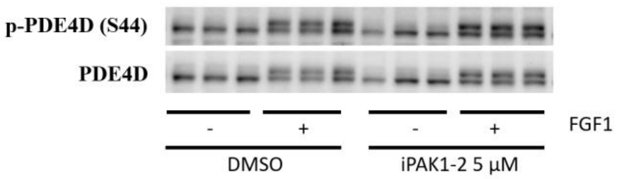

Supplemental Figure 2

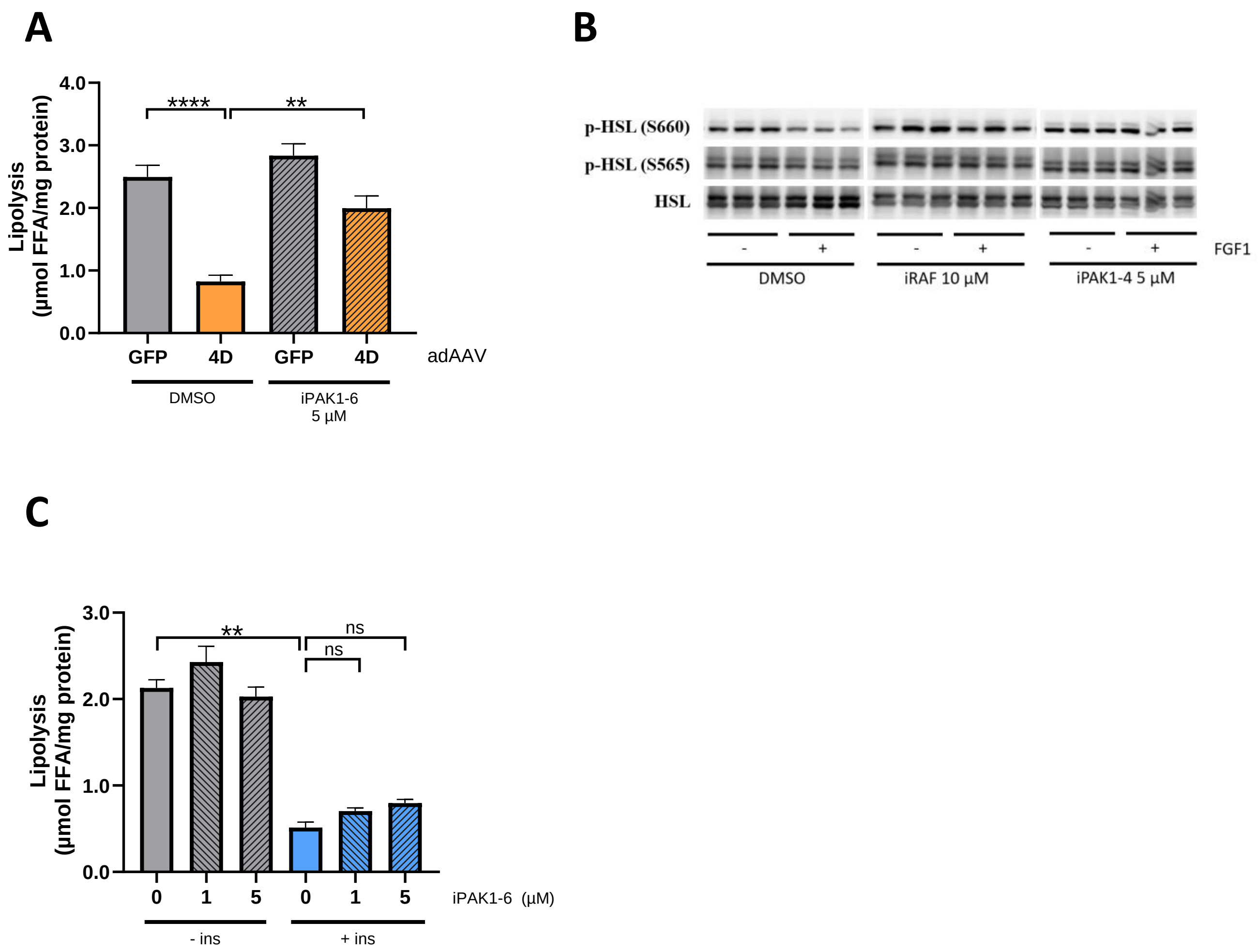

Supplemental Figure 3

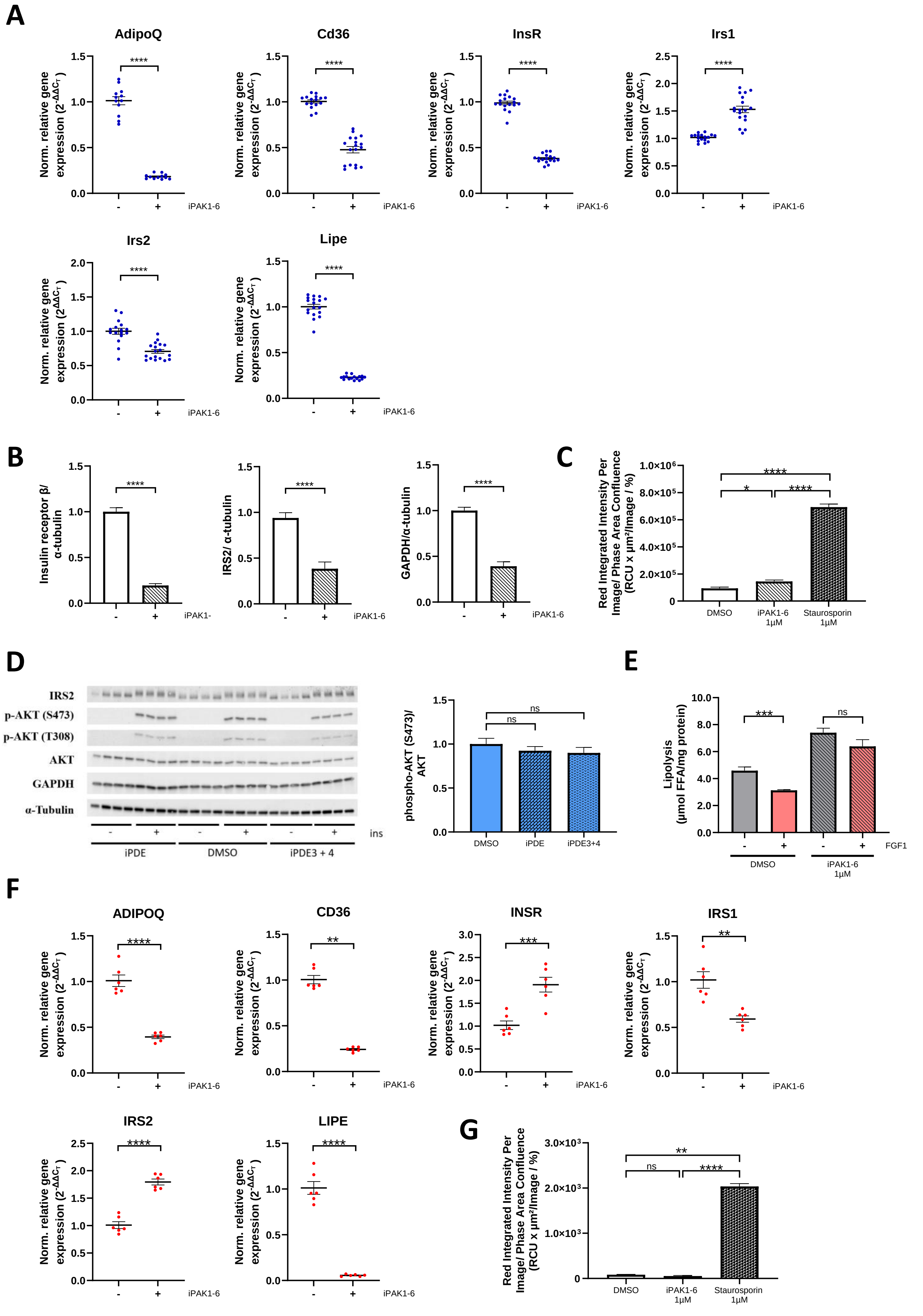

Supplemental Figure 4
